# Cancer Invasion: Patterns and Mechanisms

**Published:** 2015

**Authors:** N. V. Krakhmal, M. V. Zavyalova, E. V. Denisov, S. V. Vtorushin, V. M. Perelmuter

**Affiliations:** Siberian State Medical University, Moskovskiy Trakt, 2, 634050, Tomsk, Russia; Tomsk Cancer Research Institute, Kooperativny Pereulok, 5, 634050, Tomsk, Russia; Tomsk State University, Prosp. Lenina, 36, 634050, Tomsk, Russia

**Keywords:** cancer, invasion, cell migration, collective cell migration, individual cell migration

## Abstract

Cancer invasion and the ability of malignant tumor cells for directed migration and metastasis have remained a focus of research for many years. Numerous studies have confirmed the existence of two main patterns of cancer cell invasion: collective cell migration and individual cell migration, by which tumor cells overcome barriers of the extracellular matrix and spread into surrounding tissues. Each pattern of cell migration displays specific morphological features and the biochemical/molecular genetic mechanisms underlying cell migration. Two types of migrating tumor cells, mesenchymal (fibroblast-like) and amoeboid, are observed in each pattern of cancer cell invasion. This review describes the key differences between the variants of cancer cell migration, the role of epithelial-mesenchymal, collective-amoeboid, mesenchymal-amoeboid, and amoeboid- mesenchymal transitions, as well as the significance of different tumor factors and stromal molecules in tumor invasion. The data and facts collected are essential to the understanding of how the patterns of cancer cell invasion are related to cancer progression and therapy efficacy. Convincing evidence is provided that morphological manifestations of the invasion patterns are characterized by a variety of tissue (tumor) structures. The results of our own studies are presented to show the association of breast cancer progression with intratumoral morphological heterogeneity, which most likely reflects the types of cancer cell migration and results from different activities of cell adhesion molecules in tumor cells of distinct morphological structures.

## INVASIVE GROWTH AND METASTASIS AS MANIFESTATION OF CANCER MALIGNANCY

The results of numerous experimental and clinical studies of malignant neoplasms have indicated that invasive growth and metastasis are the main manifestations of tumor progression, which represent two closely related processes.

A malignant tumor is characterized by the possibility to implement such a biological phenomenon as the metastatic cascade that is a unique multi-stage “program” where cell invasion is a trigger and a key factor for further cancer progression and metastasis in distant organs and tissues. Massive metastatic lesions lead to the development of severe organ failure and, therefore, a patient’s death [1-3]. The range between “end” points of a complex invasive metastatic process –invasion of the primary tumor into surrounding tissues and the formation of metastatic foci –comprises several stages, the passage of which is strictly necessary for the successful development and subsequent progression of tumor growth: intravasation, survival and presence in the systemic circulation, extravasation with subsequent colonization of organs by tumor cells, and the formation of clinically detectable metastasis [1, 4-6]. Tumor growth is accompanied by increasing pressure on extracellular matrix structures, whereas the tissue microenvironment fights to retain its functional-anatomic integrity via increasing pressure on tumor cells. The factors limiting the growth of malignant neoplasm include the basal membrane and various components of the surrounding stroma, increased interstitial pressure, limited oxygen supply to tumor cells and the formation of active oxygen forms, hypoxia conditions, and permanent exposure to immune system cells. Given the intratumoral heterogeneity, in the struggle for survival, some tumor cells may be subjected to regression and death, while other cells, which resist powerful, counteracting microen vironmental factors, gain an aggressive phenotype and the ability of metastatic progression [7]. Invasive tumor growth is enabled by the detachment of malignant cells from the tumor mass due to a reduction in or complete loss of intercellular adhesion molecules, and, therefore, the cells gain the ability of anomalously high motility enabling penetration through the stiff structural elements of the surrounding stroma [8]. In this case, the invasion process extensively involves various molecular and cellular mechanisms that, according to published data, depend directly on another biological phenomenon – the epithelial-mesenchymal transformation, which was first described by E.D. Hay in 1995. Later, the term “epithelial-mesenchymal transition” (EMT) was put to use to clarify the reversibility of this process [9]. Currently, EMT is known to underlie the processes of embryogenesis and inflammation and regeneration of tissues and, certainly, plays a key role in the mechanisms of carcinogenesis [10, 11].

## PHYSIOLOGICAL PROTOTYPES OF INVASIVE GROWTH

Tumor cells spreading into the surrounding tissues and distant organs are known to reproduce the mechanisms and migration types characteristic of normal, non-tumor cells during physiological processes. Tumor cells, similar to normal cells, are capable of activating these mechanisms for changing their own shape, creating conditions for moving, as well as remodeling surrounding tissues to form migration pathways. The main problem is that tumor cells, in contrast to normal cells, do not have physiological “stop signals” to terminate these processes. Most likely, this leads to the establishment of the migration mechanisms and promotes the progression and spread of the tumor [12-14].

Malignant cells were found to use built-in genetic programs to implement the processes that determine invasive growth and the possibility of metastasis. For example, the movement of a single cell is observed during embryonic development and inflammation (e.g., leukocyte migration). A similar mechanism of dissemination is typical of cancer cells during tumor progression and metastasis [13].

Along with single cell migration, collective cell migration can occur when groups of firmly interconnected tumor cells are migrating [15, 16]. This type of migration indicates tissue rearrangement, underlies the processes of embryonic morphogenesis, and also is an essential component in the healing of wound surfaces [17, 18].

Therefore, the key is that malignant tumor cells extensively use the mechanisms of both collective and single cell migration as physiological prototypes in the process of invasive growth and metastasis.

## PATTERNS OF INVASIVE GROWTH

**Fig. 1:**
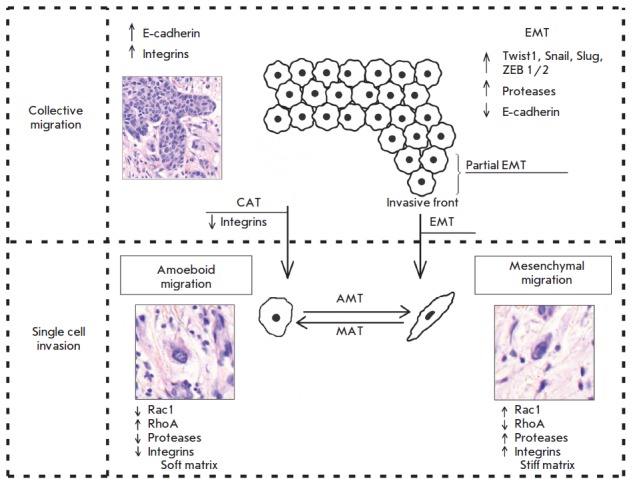
Patterns of cancer cell invasion: collective cell and individual cell migration. In collective cell migration, tumor cells exhibit high expression of E-cadherin and integrins. Epithelial-mesenchymal (EMT) and collective-amoeboid (CAT) transitions are a trigger between collective cell invasion and individual cell migration. EMT involves activation of transcription factors, such as TWIST1, Snail, Slug, ZEB1/2, a decrease in E-cadherin expression, and an increase in protease activity. During EMT, tumor cells acquire the mesenchymal phenotype, detach from the tumor mass, and migrate by the mesenchymal mechanism. In contrast, the partial EMT that is specific to the tumor invasive front means that tumor cells retain cell-cell adhesion but already possess migratory ability. This tumor cell phenotype was named the “epithelial-mesenchymal” phenotype. In CAT, which takes place when β1 integrins are down-regulated, tumor cells detach from the tumor mass and move by the amoeboid mechanism. Amoeboid migration involves a decrease in protease and integrin expression and changes in the activity of GTPases – inhibition of Rac1 and activation of RhoA. This movement type occurs in the loose/soft extracellular matrix. In contrast, mesenchymal migration is associated with the opposite phenotype and predominates in the dense/stiff matrix. These two movement types are highly plastic and can convert to each other, depending on the extracellular matrix type and intracellular regulation. Thereby, the mesenchymal-amoeboid (MAT) and amoeboid-mesenchymal (AMT) transitions are suggested [1, 13, 22, 47, 68, 73, 74]

At present, based on a complex of certain morphological and molecular genetic parameters, two fundamentally different patterns of invasive growth are distinguished: collective (group) cell migration and single cell migration (individual migration: *Fig. 1*) [1, 2, 15, 19, 20]. In this case, the migration type is largely determined by tissue microenvironment features and depends on molecular changes in tumor cells [21].

Determination of the invasion mechanism used by single migrating cells during migration is a complex task. Unfortunately, studies examining this issue at the molecular and morphological levels are few in numbers and mostly were carried out *in vitro *using specific cell lines [22].

However, now, there is considerable increase in the number of studies that demonstrate increasing interest in research into the molecular genetic features of tumor cells that determine the main differences between the mesenchymal and amoeboid types of cell movement during individual migration, as well as collective migration.

**Collective migration**

Collective migration is characterized by the migration of whole groups of cells interconnected by adhesion molecules and other communication junctions (*Fig. 1*). It should be noted that this is the main feature of this type of invasion, since the underlying cellular mechanisms are the same key processes that largely determine single cell migration [15, 20, 23, 24].

Collective cell migration has been observed in the development and progression of breast and endometrial cancer, prostate cancer, colorectal cancer, largecell lung carcinoma, rhabdomyosarcoma, melanoma, as well as most squamous cell carcinomas [1, 17, 20, 25, 26].

In the case of collective migration, cancer cells, being a part of the tumor mass or detaching from it in the form of multicellular groups, penetrate into the surrounding tissues and form thin short chords, clusters, stripes and wide fields, as well as structures with lumen, that indicate a wide variety of structural elements involved in tumor invasion [1, 2, 15, 20, 27].

As already mentioned, collective migration is characterized by the migration of whole cell groups interconnected by cadherins and intercellular gap junctions. A moving cell group has a “leading edge” or “leading front” that uses integrins and proteases (*Fig. 1*). Researchers indicate clear differences in the expression of genes and the morphology between the “leader” cells forming the leading edge and the “follower” cells that are located behind them, at the “trailing edge.” The “leaders” in the cell shape often resemble mesenchymal cells and are characterized by a less pronounced ordering and structural organization, while the “followers” tend to form more tightly packed, rosette-like tubular structures with tight intercellular contacts [17, 28].

In the case of collective migration, tumor cells form protrusions (pseudopodia) at the leading edge, use integrins to form focal contacts with the actin cytoskeleton, and perform proteolytic degradation of the extracellular matrix, creating a space for invasion of the tumor tissue and extensively involving the actin-myosin contractile apparatus in the process to ensure successful migration [15, 20].

The differences in the polarity of collectively migrating cell groups are due to the features of expression of surface receptors, such as CXCR4 and CXCR7 chemokine receptors, in the “leader” cells [29]. The growth factors and chemokines produced by stromal cells and a diffusion gradient provide extracellular induction of cell polarization. Involvement of chemokines, such as SDF1 (CXCL12), the fibroblast growth factor (FGF), and the transforming growth factor β (TGF-β), in these processes has been under discussion [17, 30].

Much is known about the involvement of TGF-β in carcinogenesis, with its role being twofold. Taylor *et al*. [31] have drawn attention to the fact that TGF-β, which acts in the epithelial cells of the mammary gland as a potent tumor suppressor at the early stages of cancer, can affect tumor development via interaction with oncogenic cytokines. Increased expression of TGF-β has been associated with the progression of tumor, which has often been observed, e.g., at the later stages of breast cancer [32, 33]. The role of TGF-β in epithelial-stromal migration during tumor progression has not been studied sufficiently. TGF-β is supposed to be a key regulator of the interactions between the tumor and stroma, which promotes collective cell migration in breast cancer [34].

It has been established that leader cells express podoplanin [2], a transmembrane glycoprotein that is expressed under normal conditions in kidney podocytes, type 1 lung alveolar cells, skeletal muscle cells, placenta, etc. Podoplanin expression in breast cancer cells induces cell migration and invasion with the formation of filopodia and simultaneous retention of Ecadherin expression [2, 35].

Data have been reported indicating that collectively migrating cancer cells can use the ability of adjacent mesenchymal cells to modify the structure of the matrix and rebuild it, and then follow in their “footsteps.” In *in vitro *experiments, the introduction of fibroblasts in the culture induces collective tumor cell migration to the underlying matrix in the form of chains. Therefore, fibroblasts are a “guide” for invading tumor cells, remodeling the surrounding extracellular matrix to pathways with thick collagen bundles on the sides and a lack of a matrix in the center [36, 37].

LIM-kinase, a member of one of the protein families, plays a role in the development of collective migration by tumor cells. This protein is known to be involved in the regulation of developing invadopodias, which are structures typical of malignant tumor cells and responsible for the destruction of the surrounding extracellular matrix. Excessive activation of LIM-kinase is displayed in breast cancer. Breast tumor cells with suppressed expression of the LIM-kinase gene lose their ability to invade due to the loss of their ability to disrupt the extracellular matrix [38, 39].

**Single cell invasion or individual cell migration**

Such a type of invasive growth as single cell invasion is distinguished based on the detection, during morphological analysis, of individual tumor cells that invade the surrounding tissues independently of each other [2]. In this type of tumor invasion, single cell migration can occur via two different movement types: mesenchymal and amoeboid [1, 2, 15, 22]. It should be noted that a number of researchers point to the possibility of a “shift” from one type of migration to the other (from mesenchymal to amoeboid and vice versa, *Fig. 1*) in the case of single cell invasion. These transitions usually occur upon changes in the activity of certain cell molecules when tumor cells have to adapt to the peculiarities of the microenvironment [22, 40].

**Mesenchymal (fibroblast-like) cell migration**

The mesenchymal mechanisms of invasive cell growth, in contrast to the amoeboid type of migration, are characterized by the occurrence of more complex processes and a need for the involvement of a larger number of cellular molecules in its implementation (*Fig. 1*).

This type of migration is typical of keratinocytes during reparative regeneration, endotheliocytes, smooth muscle cells, and fibroblasts. Since malignant cells, which use the mesenchymal type of movement, lose epithelial polarity and gain an elongated spindle shape, which resembles the fibroblast shape, invasion of this type is also called “fibroblast-like” migration [1, 2, 22, 23, 41]. Mesenchymal invasion has been detected during the development of melanoma, fibrosarcoma, glioblastoma, and other malignancies [1, 42-44].

Most of the cancer cells that detach from the tumor mass and invade the surrounding tissues are known to undergo certain changes, acquiring the morphological properties and a phenotype typical of mesenchymal cells [2, 15]. This transformation of a malignant epithelial cell, which is related to the emergence of new molecular and morphological features in the cell, was called the “epithelial- mesenchymal transition.” As already mentioned, this biological phenomenon was first described by E.D. Hay in 1995 [9]. Today, the existence of the phenomenon is supported by the results of a large number of studies that have investigated the mechanisms of invasion and metastasis of malignant tumors [1, 2, 15, 45]. The mesenchymal mechanism of invasion is believed to be the consequence of EMT, when active dedifferentiation of a malignant epithelial tumor occurs, and multicellular groups start to divide into single tumor cells, gaining a mesenchymal phenotype [13].

A number of researchers have stressed that tumor cells during the mesenchymal type of migration go through a number of specific sequential steps that constitute a five-stage model of migration. This cycle includes the following changes: 1) formation of a protrusion on one of the cell poles – a lamellipodia or a filopodia produced by contractions of the actin cytoskeleton under the control of small GTPases Rac1 and Cdc42 with rapid involvement of integrins of the β1 family; 2) occurrence of focal adhesion with the involvement of integrins β1 and β3 at the contact site between the extracellular matrix and the cell; 3) assembly of focal contacts, which is based on integrin-mediated interactions, and activation of proteolytic enzymes (matrix metalloproteinases, serine and threonine proteases, cathepsins) at the “cell-matrix” interface that leads to the destruction and remodeling of the surrounding extracellular matrix; 4) a change in the actin cytoskeleton polarization under myosin II-mediated control, the occurrence of cell body contractions; and 5) “pulling” the trailing edge toward movement through the newly formed defects in the matrix structure [1, 13, 22]. Since the cells which use the fibroblast-like mechanism of invasion follow the described migration steps, their speed of movement is low: about 0.1– μm/min [1, 22, 40].

The possibility of proteolysis and remodeling of tissue structures explains the fact that mesenchymal movement of a tumor cell is accompanied by minor changes, compared to amoeboid migration, in the cell’s shape and by minimal deformation of the nucleus [46]. Of clear interest are the results of studies that indicate that the behavior of tumor cells during individual migration depends on the surrounding matrix’ stiffness. For example, the mesenchymal or proteolytic model of migration dominates under conditions of a “stiff” (“dense”) surrounding matrix. The high migration efficiency of a single cell using the mesenchymal mechanism in dense tissues is explained by proteolysis due to the secretion of various proteases and by the ability to form focal contacts with stromal elements [47, 48].

Therefore, it is worth noting that the key points of the fibroblast-like mechanism of invasive growth are strong adhesion forces on both poles of the cell as well as between cells and extracellular matrix components, pronounced expression of integrins (β1 and β3 families), proteolysis with destruction and subsequent remodeling of tissues with the formation of defects in the matrix structure, and movement of a single cell or cell chains through the defects. The nucleus deformation is minimal, and a slow rate of cell migration is observed.

Based on the suppression of the expression of the relevant genes using small interfering RNAs, the specific activity of GTPases Rac1 and Cdc42 was demonstrated to be the characteristic feature of the mesenchymal type of invasion. Suppression of GTPase Rac1 through signaling activation of GTPase RhoA and its effector, ROCK kinase, leads to blockage of the mesenchymal migration of tumor cells [49-52].

**Amoeboid cell migration**

The amoeboid mechanism of invasive growth is the most primitive and, at the same time, the most efficient mode of migration of single tumor cells. In all of its features, it is similar to the behavior and movement of a single-celled organism, such as the amoeba *Dictyostelium discoideum* [40, 53].

The use of antibodies that block integrins or protease inhibitors in clinical trials leads to the emergence of tumor cells with the amoeboid type of migration [1]. Similar results were obtained in studies of malignant tumors *in vivo*. A relationship between the application of drugs on the basis of matrix metalloproteinase inhibitors in cancer therapy and progression of the tumor process was established. The explanation of this relationship became possible only after the identification of tumor cells capable of amoeboid migration [54]. These data most likely indicate that, under conditions of a reduction in or complete loss of their ability to spread to the surrounding tissues using the main molecules that perform adhesion and destruction of the extracellular matrix, tumor cells turn to the amoeboid mechanism of invasion, which becomes the only and most effective mode of migration.

This type of migration has been described in circulating stem cells, leukocytes, and certain types of tumor cells [2, 14]. According to Zijl *et al*., the amoeboid type of invasive growth has been observed in breast cancer, lymphoma, small cell lung cancer and prostate cancer, and melanoma [1, 42, 55].

In the case of amoeboid migration, malignant tumor cells have been demonstrated to have a round or elliptical shape (*Fig. 1*) [1, 22, 23, 40]. Amoeboid cells are characterized by fast deformability, adaption of their shapes to existing structures of the surrounding extracellular matrix, and penetration through them via narrow spaces in a compressed form. Movement and relocation are carried out through successive high-speed cycles of expansion and contraction of the cell’s body with the development of “bleb-like” protrusions of the cell membrane [22, 56-58]. These blebs allow the cell to investigate the microenvironment to find the most suitable route of movement to bypass various obstacles, whereby tumor cells are capable of moving through narrow gaps in the extracellular matrix [1, 2, 15, 22]. Developing changes in the cell shape are generated by the cortical actin cytoskeleton that is, in turn, controlled by small GTPase RhoA and its effector, ROCK kinase [1, 2, 15, 59]. This GTPase belongs to the superfamily of small GTP hydrolases, whose members play key roles in the amoeboid type of invasion, since they are involved in signal transduction and, thereby, in the regulation of a wide variety of processes occurring in the cell, including reorganization of the actin cytoskeleton during migration [51, 60, 61].

It is worth noting that migration through the amoeboid mechanism of invasion is accompanied by changes not only in the cell shape, but also in the shape of the nucleus and its orientation and position relative to other internal organelles. The nucleus, which is the largest and stiffer, compared to the surrounding cytoskeleton, organelle, is mechanically firmly stabilized by an extensive network of structural proteins, and, for this reason, its shape, most likely, often does not undergo significant changes. However, the amoeboid type of migration is characterized by the most pronounced nucleus deformation, caused by the lack of proteolytic degradation of the surrounding matrix. Since tumor cells have to move through narrow spaces and pores, the nucleus in this case also occurs in a maximum compressed state [46, 62, 63]. It is assumed that, like the amoeboid movement of leukocytes, nuclei inside single migrating tumor cells move forward toward the leading edge [46].

In contrast to the mesenchymal movement, amoeboid or a non-proteolytic model of migration prevails when the surrounding matrix is characterized by relatively low stiffness (“soft” matrix). For example, amoeboid migration of tumor cells in the lymphatic and circulatory systems is considered as migration in a soft matrix [47, 48].

Condeelis and Segall [64] elucidated some features of cell migration on the example of two different tumor lines, MTC and MTLn3, under *in vitro *and *in vivo* conditions. MTLn3 cells that have a high metastatic potential and migrate probably by the amoeboid mechanism of invasive growth are characterized by a higher level of expression of epidermal growth factor receptors (EGFRs) than MTC cells with a low metastatic potential. Their migration is associated with the presence of blood vessels and collagen-containing fibers in the surrounding matrix. Tumor cell chemotaxis towards blood vessels is believed to be mediated by the signaling pathways of EGFR [64].

The amoeboid mechanism of invasion has a number of distinctive features. It is characterized by a weak interaction between cells and the surrounding matrix, as well as a lack of or weak focal contacts. The possibility to retain the rapid and non-focal assembly of receptors at the sites of cell contacts with the extracellular substrate has been noted. Integrins are not important in this type of invasive growth. Important aspects are the absence of proteolysis at the sites of cell-matrix interactions and the lack of expression of proteolytic enzymes that destroy the extracellular matrix [1, 2, 15, 62, 65]. *In vitro *studies have demonstrated that, in the case of an amoeboid type of invasive growth, it is likely due to these properties that tumor cells are capable of moving at the highest speed in cultures (20 μm/min) [1, 20, 21].

**Amoeboid-mesenchymal and mesenchymal-amoeboid transitions**

We have already noted the existence of a degree of plasticity and the possibility of a “shift” from one migration type to the other (from the mesenchymal type to the amoeboid one and *vice versa*) upon individual cell invasion. These events are apparently due to the appearance of changes in the activity of certain cell molecules and the need to adapt to tissue microenvironment conditions (*Fig. 1*).

These changes are described as amoeboid-mesenchymal and mesenchymal-amoeboid transitions [2, 22]. Tumor cells using the mesenchymal type of migration can be changed in a certain way and shift to the amoeboid type of movement under conditions of a weakened signal and mechanical pathways that are directly involved in the stabilization of the interactions between extracellular matrix structures and malignant cells [22, 40, 47, 66]. However, the available data were obtained primarily by means of experiments. The following mechanisms leading to the transition of cells from the mesenchymal to the amoeboid type of invasive growth (mesenchymal-amoeboid transition) have been described: 1) reduction in or complete abolition of pericellular proteolysis due to application of protease inhibitors; 2) reduction in the activity of integrin receptors and their interactions with surrounding stromal elements by their antagonists; 3) increase in and stabilization of the activity of small GTPase RhoA and its ROCK effector [16, 40]. A study by S. Berton’s group provided an interesting fact indicating that the p27 protein, despite a great variety of functions, plays an important role in the control of cell motility. In particular, a lack of this protein under *in vitro *conditions induces the mesenchymal-amoeboid transition in cells in a 3D matrix [66].

Some authors studying the mechanisms of invasive growth upon individual cell migration indicate the possibility of an amoeboid-mesenchymal transition that is the reverse process to the mesenchymalamoeboid transition. There is a hypothesis according to which the mechanism of amoeboid-mesenchymal transition most likely relies on the same molecular basis, and that the only reliable process that determines the possibility of the described transformation is an imbalance in the activity of members of the small GTPase family and predominance of the Rac activity over the RhoA activity. It should be noted that the mechanisms that could underlie the described changes remain unclear [47].

## COLLECTIVE-INDIVIDUAL TRANSITIONS

Tumor cells within a single tumor can simultaneously move both collectively and individually. In this case, the transition from individual to collective migration is an important step towards increasing the invasive and metastatic potential of malignant neoplasms. For example, breast tumor cells detached from the solid mass gain the ability to invade lymphatic vessels [26]. Currently, two mechanisms are distinguished: epithelial-mesenchymal and collective-amoeboid transitions by which individually migrating tumor cells are produced (*Fig. 1*) [13, 67]. In turn, the latter, in particular cells that have undergone EMT, are capable under certain conditions of gaining an epithelial phenotype and forming tumor multicellular complexes. This phenotype inversion was called the “mesenchymal-epithelial transition” [15, 17].

**Epithelial-mesenchymal transition**

Lately, there has been vigorous discussion of the epithelial- mesenchymal transition as a mechanism during which the tumor cell detaches from the epithelial layer and gains motility (*Fig. 1*), the so-called “locomotor phenotype,” which promotes invasive growth and metastasis [68-71]. The development of this process as a key factor of cancer progression was shown *in vitro* using specific tumor lines as well as experimental models; however, establishment of the EMT development and identification of tumor cells and their main characteristics under *in vivo *conditions is a complex task [72].

EMT is the basis of many processes of morphogenesis [71]. It is believed that under normal conditions (during embryogenesis) EMT can be induced by the HGF (hepatocyte growth factor) secreted by fibroblasts. HGF binds to specific c-Met receptors located on the membrane of epithelial cells. The binding to receptors activates a signaling pathway involving some proteins of the small GTPase system (Cdc42, Rac, RhoA, RhoC) that regulate the intensity of actin microfilament polymerization and the contractility of actin-myosin filaments, which determines the intensity of lamellipodia formation and tension of the matrix-attached cell. In this case, there is significant rearrangement of the whole actin-myosin cytoskeleton and loss of E-cadherin intercellular contacts. During carcinogenesis, epithelial cells are subjected to a morphological transformation that is phenotypically similar to EMT but develops in the absence of the relevant HGF ligand. This transformation in malignant tumors can be induced by transfection of various oncogenes. During transformation, tumor cells can leave the epithelial layer and move like fibroblasts, thereby gaining the ability of invasion and metastasis [73].

During EMT, the following major events occur: malignant epithelial cells lose their apical-basal polarity due to disruption in tight intercellular junctions and loss of cellular adhesion molecules (such as E-cadherin and integrins); the cellular actin cytoskeleton is changed and subjected to remodeling with the formation of stress fibers that are collected in certain cell parts near the cell membrane, where specific cellular protrusions begin subsequently to form; degradation of the underlying basal membrane of the epithelium occurs, which results in the fact that tumor cells lacking intercellular contacts become capable of invasive growth and penetration into the surrounding stromal matrix and begin active migration [69, 71].

EMT was found to be rarely equally pronounced in the entire tumor tissue. More likely, this process is characterized by a varying intensity of the transition of cells from the epithelial to the mesenchymal phenotype. In this regard, some researchers describe the socalled partial EMT, in which most cells in the invasive front are involved (*Fig. 1*). Partial EMT is a state when cells have already gained the properties necessary for successful migration, but continue to retain cell-cell contacts. This phenotype was called the hybrid “epithelial- mesenchymal” phenotype and was linked to the features characteristic of collectively moving tumor cells [69, 74, 75].

Taddei *et al*. have indicated that EMT develops due to the induction of programs associated with the activation of key transcription factors, such as TWIST1, Snail, Slug, and ZEB1/2 [76, 77]. This results in disruption in strong cadherin junctions and activation of polar cell migration and proteolysis of extracellular matrix components by various secreted proteases, with the functions of integrin receptors being retained [10, 17, 77, 78]. The role of the transcription factor Prrx1, which determines the ability of breast cancer cells for invasive growth, was experimentally established [79].

It was shown that ZEB1 and ZEB2 proteins with a zinc finger domain are able to directly bind to promoters, thereby inducing the expression of mesenchymal marker genes and suppressing the expression of E-cadherin and other epithelial markers [80, 81].

Similarly, Snail and Slug are able to suppress the expression of the E-cadherin gene via direct binding to its promoter, as well as production of epithelial proteins such as desmoplakin and claudin, and activate the expression of vimentin and matrix metalloproteinases, thereby increasing cell migration [82]. A team of researchers led by Sanchez-Tillo found that the transcription factor Snail does not occur in normal epithelial cells and that its detection in cells of the tumor invasive front can be considered as a predictor of poor survival of cancer patients [83]. It is believed that ZEB1/2, Snail, and Slug are induced by TGF-β, inflammatory cytokines, and hypoxia [84].

**Collective-amoeboid transition**

Based on experimental data, a number of researchers indicate the possible existence of a so-called collective- amoeboid transition (*Fig. 1*), when tumor masses invading surrounding tissues in the form of collective multicellular groups dissociate into single migrating cells that use the amoeboid movement [40]. This event has been shown to become possible with the application of inhibitors of integrin receptors of the β1 family, since these molecules play a key role both in the formation of cell-cell contacts and in the interactions between tumor cells and surrounding tissue components [16, 40, 85].

**Mesenchymal-epithelial transition**

There are actually no studies devoted to the investigation of the mechanisms underlying the mesenchymal- epithelial transition. However, the possibility of such a phenomenon is recognized. In this case, it is said that often, e.g. in breast and prostate cancer, the tissue structure in distant metastatic foci is similar to the primary tumor structure [15, 86]. According to Friedl and Gilmour [17], several assumptions can be made based on these data. First, invasion and metastasis can occur without EMT. Second, detection of single disseminated cells during a routine pathologic examination of tumor tissue samples seems to be a rather complex task, and identification of these cells during EMT is actually impossible. And, third, tumor cells temporarily use the EMT mechanisms for intravasation and spread to distant organs and tissues, where they return to the epithelial phenotype. This transformation is described as the mesenchymal-epithelial transition (MET) [15, 17]. MET has been induced experimentally, and individually moving cells formed multicellular complexes, but the molecular mechanisms of MET under physiological conditions remain unknown [17]. Nguyen *et al*. [5] demonstrated that the selective inhibitor PD173074 of the fibroblast growth factor receptor 1 (FGFR1) inhibits the MAPK signaling pathway regulating the activity of the AP-1 protein, which, in turn, induces the development of MET. Investigation of the possibility of using the PD173074 inhibitor as a drug, which was conducted on specific tumor cell lines, revealed a distinct suppression of tumor growth, migration ability, and invasion. In this case, a decrease in the expression of *Snail *and the matrix metalloproteinase 3, 10, 12 and 13 genes and an increase in the expression of the E-cadherin gene were observed [5].

## CLASSIFICATION OF INVASIVE GROWTH TYPES ON THE EXAMPLE OF BREAST CANCER

For many years, our research team has studied the features of breast cancer progression depending on intratumoral heterogeneity. Particular attention has been paid to the phenotypic diversity of the primary tumor in invasive carcinoma of no special type, which accounts for the bulk (80%) of all histological types of breast cancer.

**Fig. 2:**
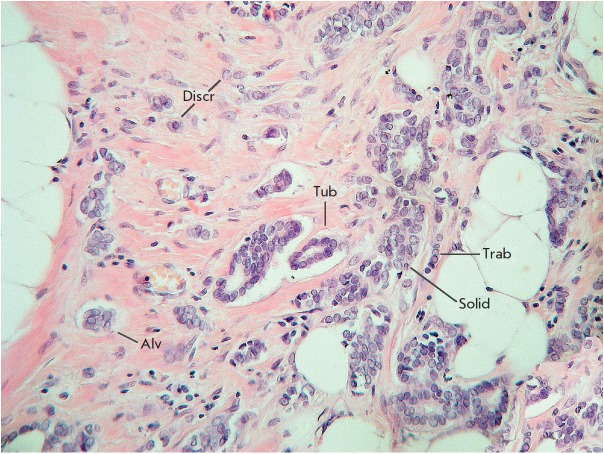
Intratumoral morphological heterogeneity in invasive breast carcinoma. Diversity of invasive growth of breast cancer is shown, which can be classified into five main morphological structures: alveolar (Alv), trabecular (Trab), tubular (Tub), solid (Solid) structures, and discrete groups of tumor cells (Discr). Hematoxylin and eosin staining. Magnification of 200x

Despite the considerable structural diversity of the primary breast tumor, five main types of morphological structures can be distinguished: alveolar, trabecular, tubular and solid structures, and discrete groups of tumor cells (*Fig. 2*). The alveolar structures are tumor cell clusters of round or slightly irregular shape. The morphology of the cells that form this type of structures varies from small cells with moderate cytoplasm and round nuclei to large cells with hyperchromatic nuclei of irregular shape and moderate cytoplasm. The trabecular structures are either short, linear associations formed by a single row of small, rather monomorphic cells or wide cell clusters consisting of two rows of medium-sized cells with moderate cytoplasm and round normochromic or hyperchromatic nuclei. The tubular structures are formed by a single or two rows of rather monomorphic cells with round normochromic nuclei. The solid structures are fields of various sizes and shapes, consisting of either small cells with moderate cytoplasm and monomorphic nuclei or large cells with abundant cytoplasm and polymorphic nuclei. Discrete groups of cells occur in the form of clusters of one to four cells with variable morphologies [87, 88].

According to the data accumulated to date, it may be assumed that different morphological structures of breast tumors correspond to certain types of invasion. Therefore, alveolar, trabecular, and solid structures that are characterized by the presence of cell-cell contacts may be referred to morphological manifestations of collective migration, while discrete groups of tumor cells may be referred to manifestations of individual migration. Interestingly, the first batch of data obtained in a study of the expression of cell adhesion genes fully confirms this hypothesis. For example, there was a decrease in the activity of the genes of cadherins, which are responsible for cellcell contacts, in the order: solid – alveolar and trabecular structures – discrete groups of tumor cells. In this case, the number of expressed genes of integrins involved in the adhesion of tumor cells to the extracellular matrix was reduced in the order: solid and alveolar – trabecular structures – discrete groups of tumor cells [89].

## TYPES OF INVASIVE GROWTH IN TUMOR PROGRESSION AND THERAPY EFFICACY

Invasive growth and the development of drug resistance are related processes that play the most important role in tumor progression: in particular in metastasis. It is very likely that the same signaling pathways are involved in cell migration and the development of tumor resistance to therapy [67, 90].

Migrating tumor cells (regardless of the movement’s type) are more resistant to chemotherapy and radiotherapy than non-moving cells [90]. This is largely due to the fact that migrating cells temporarily lose their ability to divide. It is also the fact that moving tumor cells display increased activity of anti-apoptotic genes, which causes resistance to chemotherapeutic drugs aimed at induction of programmed cell death [91]. In addition, cells in the EMT state are known to also exhibit chemoresistance [92]. This drug resistance is due to induction, during EMT, of the synthesis of the ABC family proteins responsible for the efflux of chemotherapeutic drugs out of the cell. The main transcription factors that trigger EMT and, at the same time, positively regulate the activity of ABC transporters include TWIST1, Snail, etc [92-94].

Recently obtained data indicate strong association between collective migration and resistance to radiotherapy and chemotherapy [67, 90]. According to our own research, breast tumors containing both alveolar and trabecular structures, as well as demonstrating significant morphological diversity, are characterized by increased drug resistance [95, 96]. Interestingly, the contribution of the trabecular structures to chemoresistance is probably explained by the high activity of ABC transporters in tumor cells of a given morphological variant. In contrast, resistance of breast tumors containing the alveolar structure is explained by other, yet unidentified, causes [96].

Invasive growth and its phenotypic diversity are associated, both directly and through the development of drug resistance, with metastasis. Circulating tumor cells, which are responsible for the development of future metastases, are a result of the invasion and subsequent penetration of tumor cells into lymphatic or blood vessels. Not only single migrating tumor cells, but also cell groups can have the intravasation ability. There is an assumption that collective migration much more often leads to metastasis compared to individual migration. Pioneering studies in animal models have demonstrated that metastases more often form after intravenous injection of tumor clusters rather than single tumor cells [97-99]. Furthermore, circulating tumor cell clusters have been found in the blood of patients with various cancers [100, 101]. It was assumed that collective intravasation is related to the VEGFdependent formation of dilated vasculature and the accumulation of intravasated tumor clusters [102]. Furthermore, groups of tumor cells can enter circulation through damaged vessels [103] or by cooperation with cells in the EMT state and cancer-associated fibroblasts that disrupt the extracellular matrix by proteases [14, 104]. The dependence of metastasis on collective migration is confirmed by the results of our own research. For example, the presence of alveolar structures in tumors in postmenopausal breast cancer patients is associated with a high rate of lymphogenous metastasis, whereas the risk of this type of progression in premenopause females increases with an increase in the number of different types of morphological structures [87, 105]. The latter dependence is also quantitative: lymphogenous metastases were detected more frequently in the case of a larger number of alveolar structures in breast tumors [87, 106]. Furthermore, patients with alveolar structures in tumors had a low metastasis-free survival rate (our own unpublished data).

The established relationship between the alveolar structures, as one of the manifestations of collective migration, and the rate of lymphogenous and hematogenous metastasis allows us to put forth the following assumptions. Apparently, the cellular elements of the alveolar structures differ from tumor cells of other structures by a set of biological properties determining the metastatic phenotype. The clearer relationship between alveolar structures and lymphogenous metastasis in the menopausal period suggests a certain role of estrogens, including also their production *in situ*, in that tumor cells of the alveolar structures gain the metastatic phenotype through the lymphogenous pathway [107].

Therefore, the data currently available on the features of invasive growth in carcinomas of different localizations and, in particular, in breast cancer present new opportunities for the investigation of tumor progression patterns and the search for additional key parameters of prognosis and, possibly, “control” of disease progression.

## CONCLUSIONS

The significance of studies of the morphological manifestations and molecular genetic mechanisms of the invasion and metastasis of malignant tumors is not in doubt. The results of numerous studies clearly demonstrate that migration of tumor cells during invasive growth can occur both via single cells and via groups of cells. This diversity of cell migration types probably leads to the development of intratumoral heterogeneity that is represented, e.g. in breast cancer, by different morphological structures: alveolar, trabecular, and solid structures and discrete groups of tumor cells. A number of biochemical and molecular genetic mechanisms are known that enable malignant cells to invade surrounding tissues and gain the ability to spread far beyond the primary tumor site, giving rise to the development of secondary metastatic foci in distant organs and tissues. However, despite the achieved progress, there remain unexplored questions concerning a possible relationship between different types of invasive cell growth and the parameters of lymphogenous and hematogenous metastasis, the features of disease progression, as well as the efficacy of the chosen therapy. A solution to these problems could be of great help in determining the disease prognosis and, possibly, developing new approaches to the management of cancer patients.

## Abbreviations

EMT: epithelial-mesenchymal transition
MET: mesenchymal-epithelial transition
GTPases: guanosine triphosphatases
